# Factor structure and measurement invariance of the Chinese version of the Center for Epidemiological Studies Depression (CES-D) scale among undergraduates and clinical patients

**DOI:** 10.1186/s12888-021-03474-x

**Published:** 2021-09-23

**Authors:** Lishui Niu, Jiayue He, Chang Cheng, Jinyao Yi, Xiang Wang, Shuqiao Yao

**Affiliations:** 1 Medical Psychological Center, The Second Xiangya Hospital, Central South University, Changsha, 410011 Hunan China; 2 Medical Psychological Institute of Central South University, Changsha, 410011 Hunan China; 3 National Clinical Research Center for Mental Disorders, The Second Xiangya Hospital, Central South University, Changsha, 410011 Hunan China

**Keywords:** CESD, Depression, Factor structure, Measurement invariance

## Abstract

**Background:**

The Center for Epidemiologic Studies Depression scale (CESD) was widely used for screening of depressive symptoms. The purpose of the current study was to investigate the factor structure and measurement invariance of the CESD across genders and groups in a sample of Chinese undergraduates and clinical patients.

**Methods:**

Participants included 3093 undergraduates from the Hunan province and 336 patients from psychological clinics. The structure of the CESD scale was analyzed by confirmatory factor analysis (CFA). Multiple sets of CFAs were used to test measurement invariance across genders among undergraduates and clinical patients. Internal consistency reliability was also evaluated.

**Results:**

The five-factor model achieved satisfactory fit (in the undergraduate sample: WLSMVχ^2^ = 1662.385, df = 160, CFI = 0.973, TLI = 0.968, RMSEA = 0.055; in the clinical patients: WLSMVχ^2^ = 502.089, df = 160, CFI = 0.962, TLI = 0.955, RMSEA = 0.072). The measurement invariance of the five-factor model across genders was supported fully assuming different degrees of invariance. The CESD also showed acceptable internal consistency.

**Conclusion:**

Due to its sound structure and measurement invariance, the five-factor model of the CESD is best suited for testing in Chinese mainland college students and clinical patients.

## Background

Depression is a common mental illness, and can lead to functional impairment, disability, and even suicide [1, 2]. According to the World Health Organization (WHO), depression has become the fourth most common mental illness in the world, and it has been named the “disease of the century” [3]. Undergraduates are at a high-risk of depression due to pressures of employment, interpersonal communication, social pressures, lack of emotional feedback, and homework [4, 5]. A meta-analysis of studies published between January 1990 and October 2010 on depression among undergraduates and medical students reported an average depression prevalence rate of 30.6% worldwide [6]. Suicide and self-mutilation caused by depression are common in undergraduates and may be increasing [7].

The Center for Epidemiologic Studies Depression scale (CES-D), developed in 1977 by Radloff [8], is one of the most widely used self-report scale to assess depressive symptoms. The scale items cover the major components of depressive symptoms and was designed to measure the current level of depression [9]. Thus, the CESD has been widely used in research on children and adolescents and elderly populations, the physically ill and the mentally ill populations [9–13]. The CESD has shown good reliability (Cronbach α = 0.70–0.95, r_test-retest_ = 0.71–0.85) and good validity in different countries [13–15]. The Chinese version of the CESD has been reported to be useful for assessing depression among large adolescents and adults [9].

The original version of the CES-D consisted of 20 items, categorized into 4 factors: depressed affect (DA; seven items); somatic complaints (SC; seven items); interpersonal problems (IP; two items); and positive affect (PA; four items) [8]. However, others have proposed two [16], three [9, 17] and five [15, 18] factor models. In 2006, Shafer conducted four separate meta-analyses based on factor analysis studies of the CES-D including a total of 22,000 participants and found that the original four-factor structure was the most suitable [19]. However, another meta-analysis by Kim et al. (2011) found that the four-factor structure of the CES-D was not appropriate among Asian participants. Besides, the four-factor model has been shown to be the best fitting model across various Chinese factor analytic studies [10, 11]. However, Wang et al. found that three-factor model (depressed affect, somatic complaints and positive affect) was the best fitting model among Chinese adolescents [9]. A confirmatory factor analyses indicated that another three-factor structure (positive affect, interpersonal problems, depressive mood and somatic symptoms combined) had good fit in rural Chinese [20]. Thus, the best factor structure of the CES-D among Chinese participants has not yet been determined. It is important to confirmed the best fitted model of the CESD among different samples of China.

Based on the best fitted model, another essential issue that requires further study is whether the CESD has the same structure in different groups and whether its items have the same meaning for across different groups. Previous studies have found differences in CESD scores between male and female college students [12]. A longitudinal study found that a higher percentage of male students endured different degrees of depression compared to female students [21]. In these comparative studies, it is presumed that the measurement of the construct is comparable between male and female. However, since the meaning of items may differ for males and females, it is necessary to establish the measurement invariance of the CESD between different the twpo. Measurement invariance is defined as “a given factorial defined construct has the same measurement parameters across two or more samples (i.e. the loading, intercepts and residual matrix are equal among different groups)” [22, 23]. Without evidence of measurement invariance, it cannot concluded that group difference in depression reflected true differences between groups, as the difference may be due to the item bias of the scale [23]. A previous study has demonstrated that the measurement invariance of the CESD was acceptable across gender among non-clinical sample [9], but the result was not generalized to clinical populations.

Thus, the aims of the present study were to test the factor structure and internal consistency reliability of the CESD in undergraduates and clinical patients and to explore measurement invariance of the CESD across genders among the two samples.

## Methods

### Participants

The undergraduate participants came from the Central South University in Changshang. We recruited participants by posters and advertisements. Students who had a history of a mental disorder, a neurological disorder and intellectual disability were excluded. A total of 3158 university students were surveyed, 10 of which were excluded due to mental disorders and 55 of which were excluded due to missing data. The final sample included 3093 (57% males, 43% females), aged 18 to 22 years old [Mean = 19.5, Standard Seviation(SD) = 1.04].

The clinical samples including 353 outpatients who had been referred for the assessment and treatment in a psychological clinic of the Second Xiangya Hospital. The patients who cannot understand the questions well were excluded. A total of 336 patients finished the questions, including 139 (42%) males and 197 (58%) females, aged 16 to 33 years old (Mean = 24; SD = 5.7). The diagnoses of clinical sample were major depressive disorder(38.5%), schizophrenia(10%), obsessive-compulsive disorder(11.8%), a personality disorder(7.4%), an anxiety disorder(14.7%)and other mental disorders(16.9%)as a whole and the frequency distribution of the psychiatric disorders were 31.1%. 13.1, 13.1, 5.5, 16.4, 19.1 and 44.4%, 7.6, 10.7, 8.9, 13.3, 15.1% separately for males and females. There was significant difference between undergraduate participants and clinical samples on age (t = − 9.79, *p* < 0.01).

The data were collected by a trained psychology postgraduate researchers. All participants provided informed consent and the Ethics Committee of the Second Xiangya Hospital of Central South University approved the study. There were no significant demographic differences between participants who did not complete the CES-D and those that did in two groups.

### Measures

#### CES-d

The CES-D consists of 20 items, including 16 negative items (“I felt depressed”, “I Felt lonely”) and 4 positive items(“I was happy”, “I Enjoyed life”). The four positive affect items were inversely scored for calculating the total score. Items are structured on a 4-point from 0 (rarely; less than 1 day) to 3 (most or all of the time; 5–7 days). Higher scores on the CES-D indicate more depressive symptoms. The Chinese version of CES-D has been widely used in China and has been validated in previous Chinese studies [11, 20, 24].

#### Data analysis

##### Step 1: confirmatory factor analysis (CFA)

The CFAs were analyzed with Mplus 7.11 software to examine the best fit factor model of the CES-D. Given that items have only four response categories, the robust weighted least squares with mean and variance adjustment (WLSMV) estimator was used [23, 25, 26]. Several models fit indices were used to evaluate the goodness of fit: the Tucker-Lewis Index (TLI), the comparative fit index (CFI), and the root mean-square error of approximation (RMSEA) [9, 27]. According to the conventional guidelines, CFI, TLI ≥ .90 indicates acceptable model fit and ≥ .95 indicates adequate model fit, while RMSEA values ≤ .08 indicates acceptable model fit and ≤ .05 indicate good model fit [28, 29].

Six alternative models of the CES-D, which were good fitted in previous studies, were chosen for comparison. Model A was the original four-factor model proposed by Radloff [8]. In this model, items loaded on four factors: depressed, somatic, interpersonal, and positive. The four-factor model has been shown to be the best fitting model across various Chinese factor analytic studies. Model B is a two-factor model which included depressed affect and positive affect [12]. All negative terms are combined into the depressed affect, and the remaining positive terms form the positive affect. Recently, this model also has been verified good fitted in Chinese population. Model C was completed after Kuo’s study to analyze the factor structure of Chinese Americans and put forward a three-factor model (depressed affect, positive affect and interpersonal problems), and the results about Chinese American were superior to the three-actor model in Kuo’s study [13] Model D is another three-factor model proposed by Wang et al. which including depressed affect, positive affect and somatic complaints factors [9]. It is shown to be best fitting in Chinese adolescents. Model E and Model F are five-factor model proposed by Kim for Asian population after meta-analysis by Exploratory factor analysis (EFA) and CFA separately [15]. EFA is a data-driven approach while CFA is a model-driven approach [15]. Therefore, we include two five-factor models (model E and Model F) derived from different analytical method. Model E contains one additional factor (alienation, AI) compared to original four-factor structure. Besides, in Model F, one additional factor representing sorrow/ grief appeared that was distinct from the original depression factor. Both alienation and sorrow/grief were factors unique to the Asian population in the meta-analysis (See Table 1).

**Table 1:** Item Mapping for Tested Models

Item	Model A	Model B	Model C	Model D	Model E	Model F
1. I was bothered by things that usually don’t bother me.	SC	DA	DA	SC	DA	SC
2. I did not feel like eating; my appetite was poor.	SC	DA	DA	SC	SC	SC
3. I felt that I could not shake off the blues even with help from my family or friends.	DA	DA	DA	SC	DA	SG
4. I feel that I was just as good as other people.	PA	PA	PA	PA	PA	IP
5. I had trouble keeping my mind on what I was doing.	SC	DA	DA	SC	DA	SC
6.I felt depressed.	DA	DA	DA	SC	DA	SG
7. I felt that everything I did was an effort.	SC	DA	DA	SC	DA	SC
8. I feel hopeful about the future.	PA	PA	PA	PA	PA	PA
9. I thought my life had been a failure.	DA	DA	DA	SC	SC	DA
10. I felt fearful.	DA	DA	DA	SC	AI	DA
11. My sleep was restless.	SC	DA	DA	SC	DA	SC
12. I was happy.	PA	PA	PA	PA	PA	PA
13. I talked less than usual.	SC	DA	DA	DA	SC	SC
14. I felt lonely.	DA	DA	DA	DA	AI	DA
15. People were unfriendly.	IP	DA	IP	DA	IP	IP
16. I enjoyed life.	PA	PA	PA	PA	PA	PA
17. I had crying spells.	DA	DA	DA	DA	AI	SG
18. I felt sad.	DA	DA	DA	SC	DA	DA
19. I felt that people disliked me.	IP	DA	IP	DA	IP	IP
20. I could not get “going.”	SC	DA	DA	DA	SC	SC

*NOTE. AI* Alienation, *da* Depressed affect, *IP* Interpersonal problems, *PA* Positive affect, *SC* Somatic complaints, *SG* Sorrow/Grief. Model A:Radloff’s original four-factor model; Model B: two-factor model in which all negative items were combined into an independent factor and the remaining four positive items formed a second factor; Model C:three-factor model with depressed affect, positive affect and interpersonal problems; Model D: another three-factor model proposed by Wang et al. with depressed affect, positive affect and somatic complaints; Model E: five-factor model in Kim’s meta-analysis; Model F: another five-factor model in Kim’s meta-analysis

##### Step 2: internal consistency reliability

In the current study, Cronbach’s alphas (α), mean inter-item correlations (MIC) and McDonald’s Omega coefficient were used to evaluate internal consistency reliability. A Cronbach’s α coefficient above 0.70 (> 0.60 in some cases) was considered acceptable. An optimal range of 0.10–0.40 was set for the MIC.

##### Step 3: measurement invariance

After the most appropriate factor model was identified, Mplus 7.11 was used to analyze the model’s measurement invariance across genders. The multi-group CFA (MGCFA) was used to test the invariance with nested models. MGCFA method typically considers four different levels of measurement invariance: configural, weak (metric), strong (scalar) and strict. Configural invariance to test whether the latent variables are in the same constituents or patterns across groups (Model 1). Weak invariance based on the configural invariance results to test the relationship between the measurement index and the factor load, that is whether factor loads are equal to the groups (Model 2). Strong invariance based on metric invariance results to test whether the variable intercepts are equal between different group (Model 3). Strict invariance based on scalar invariance results to test whether the error variance are equal to different groups (Model 4) [22]. Given that tests of the change in CFI are reported as being superior to chi-square difference tests of nested models, because they are not affected by the sample size [29, 30], the current study compared nested models in consideration of CFI values. Thus, measurement invariance is considered established when two of following satisfied: the change of TLI < 0.01, the change of CFI < 0.01, the change of RMSEA < 0.015 [25].

##### Step 4: difference test

T-tests were used to explore differences between males and females and between clinical and non-clinical sample on the total CES-D score and each factor score. *P*-values < 0.05 was considered significant.

## Results

### Descriptive statistics

In the undergraduate sample, CES-D rescores anged from 20 to 68 (Mean = 32.37 (SD) = 7.81). In the clinical sample, CES-D scores ranged from 21 to 80 (Mean = 54.30 (SD) = 12.32).

### CFA of the CES-D scale based on the hypothesized model

As illustrated in Table 2, Model B、Model D and Model E fitted the data well (CFIs > 0.90, TLIs > 0.90, RMSEAs < 0.08) in the clinical sample. Model E (five-factor model) provided the best fit for the data (WLSMVχ^2^ = 502.089, df = 160, CFI = 0.962, TLI = 0.955, RMSEA = 0.072) in the clinical sample. As can be seen in Table 3, Model E also fit the data well in the undergraduate sample (WLSMVχ^2^ = 1662.38, df = 160, CFI = 0.973, TLI = 0.968, RMSEA = 0.055). For all items, the factor loadings were ≥ 0.40 and loaded significantly on the latent factors propose (*p* < 0.01; Table 4).

**Table 2:** Goodness-of-fit indices of the compared models in clinical patients

	WLSMVχ^2^	df	CFI	TLI	RMSEA(90%CI)
Model A	886.139	169	0.921	0.911	0.102(0.096, 0.109)
Model B	526.342	167	0.960	0.955	0.073(0.066, 0.080)
Model C	803.954	167	0.930	0.920	0.097(0.090, 0.104)
Model D	505.707	164	0.962	0.956	0.072(0.065, 0.079)
Model E	502.089	160	0.962	0.955	0.072(0.065, 0.080)
Model F	683.346	160	0.942	0.931	0.090(0.083, 0.097)

*Note. WLSMV* Weighted least squares with mean and variance adjustment, *df* Degree of freedom, *TLI* Tucker-Lewis index, *CFI* Comparative fifit index, *RMSEA* Root mean square error of approximation; Model A: Radloff’s original four-factor model; Model B: two-factor model in which all negative items were combined into an independent factor and the remaining four positive items formed a second factor; Model C:three-factor model with depressed affect, positive affect and interpersonal problems; Model D: another three-factor model proposed by Wang et al. with depressed affect, positive affect and somatic complaints; Model E: five-factor model in Kim’s meta-analysis; Model F: another five-factor model in Kim’s meta-analysis

**Table 3:** Goodness-of-fit indices of the compared models in undergraduates

	WLSMVχ^2^	df	CFI	TLI	RMSEA(90%CI)
Model A	2482.029	169	0.958	0.953	0.067(0.064,0.069)
Model B	2081.544	167	0.965	0.960	0.061(0.059, 0.063)
Model C	2089.192	167	0.965	0.960	0.061(0.059, 0.063)
Model D	2090.777	164	0.965	0.959	0.062(0.059, 0.064)
Model E	1662.385	160	0.973	0.968	0.055(0.053, 0.058)
Model F	3474.084	160	0.940	0.929	0.082(0.079, 0.084)

**Table 4:** Model E (Five-Factor Model) Factor Loading

Item	Undergraduate sample	Clinical sample
1. I was bothered by things that usually don’t bother me.	0.635	0.718
2. I did not feel like eating; my appetite was poor.	0.464	0.519
3. I felt that I could not shake off the blues even with help from my family or friends.	0.757	0.851
4. I feel that I was just as good as other people.	0.626	0.621
5. I had trouble keeping my mind on what I was doing	0.643	0.639
6. I felt depressed.	0.826	0.892
7. I felt that everything I did was an effort.	0.777	0.757
8. I feel hopeful about the future.	0.800	0.657
9. I thought my life had been a failure.	0.772	0.666
10. I felt fearful.	0.767	0.705
11. My sleep was restless.	0.585	0.447
12.I was happy	0.812	0.873
13. I talked less than usual.	0.625	0.595
14. I felt lonely.	0.775	0.713
15. People were unfriendly.	0.837	0.836
16.I enjoyed life.	0.830	0.816
17. I had crying spells.	0.477	0.591
18. I felt sad.	0.772	0.871
19. I felt that people disliked me.	0.873	0.920
20. I could not get “going.”	0.749	0.721

### Internal consistency reliability

In both samples, the Cronbach’s α values were > 0.8 for the whole scale and > 0.6 for each dimension (Table 5). All mean MICs were between 0.10 and 0.400 except PA subscale, IP subscale in undergraduates sample and DA subscale in clinical sample. The McDonald’s Omega coefficients were > 0.9 for the whole scale and > 0.6 for each dimension (Table 5).

**Table 5:** Cronbach’s α values, mean inter-item correlations and McDonald’s Omega of the CESD

	Undergraduate sample			Clinical sample		
	Cronbach’s α	MIC	Omega coefficients	Cronbach’s α	MIC	Omega coefficients
Total scale	0.895	0.309	0.901	0.918	0.346	0.921
DA	0.814	0.392	0.821	0.854	0.476	0.866
SC	0.611	0.291	0.629	0.671	0.336	0.676
PA	0.772	0.462	0.78	0.776	0.470	0.784
AI	0.601	0.339	0.625	0.654	0.338	0.659
IP	0.732	0.480	0.734	0.815	0.387	0.815

### Measurement invariance across genders among undergraduates and clinical patients

As the five-factor model (model E) fitted the data best in undergraduate and clinical samples, we choose the five-factor model to estimate the measurement invariance across gender.

In the undergraduates sample, the following goodness of fit indices were obtained from the configural invariance test: TLI = 0.934, CFI = 0.944, RMSEA (90% CI) = 0.043 (0.040, 0.045) (see Table 6). All indices met requirements of configural invariance. Thus, the configural invariance was established and the model was used as baseline model for the next analysis. To verify weather factor loads are equal across gender, the weak invariance was set based on the baseline model. All indices met requirements of weak invariance (see Table 6). In addition, the ∆CFI, ∆TLI, and △RMSEA (0.000, 0.002, and − 0.001, respectively) were all less than 0.01. On the basis of previous steps, the strong invariance was set. All requirements for the goodness of fit indices for the strong invariance test were met (see Table 6). In addition, ∆CFI, ∆TLI, and △RMSEA (− 0.006, − 0.003, and 0.001, respectively) were all less than 0.01. The strict invariance was set on the basis of the third step. All indices of the strict invariance test were less than 0.01 (∆CFI = 0.000, ∆TLI = 0.003, and △RMSEA = − 0.001) and therefore, strict invariance was established in undergraduate sample (see Table 6).

**Table 6:** Measurement invariance of the CESD across gender

Model	χ^2^	df	CFI	TLI	RMSEA(90%CI)	ΔCFI	ΔTLI	ΔRMSEA
*undergraduate sample*								
Model 1	1224.497	320	0.944	0.934	0.043(0.040,0.045)	–	–	–
Model 2	1247.502	335	0.944	0.936	0.042(0.039,0.044)	0.000	0.002	−0.001
Model 3	1353.658	350	0.938	0.933	0.043(0.041,0.045)	−0.006	− 0.003	0.001
Model 4	1374.808	370	0.938	0.936	0.042(0.040,0.044)	0.000	0.003	−0.001
*clinical sample*								
Model 1	722.652	320	0.956	0.948	0.079(0.071,0.086)	–	–	–
Model 2	723.161	334	0.958	0.952	0.076(0.068,0.083)	0.002	0.004	−0.003
Model 3	795.027	370	0.954	0.953	0.075(0.068,0.082)	−0.004	0.001	−0.001
Model 4	760.657	390	0.960	0.961	0.068(0.061,0.076)	0.006	0.008	−0.007

*Note:* Model 1: morphological invariance; Model 2: metric invariance; Model 3: strong invariance; Model 4: strict invariance

In the clinical sample, in the configural invariance test, various parameters were allowed to be freely estimated, and the following fitting indicators are obtained in clinical sample: TLI = 0.948, CFI = 0.956, RMSEA (90% CI) = 0.079 (0.071,0.086). Fitting index met the requirements of the survey and the baseline model was established. Based on the baseline model, the changes of CFI, TLI and RMSEA(CFI < 0.010, TLI < 0.010, RMSEA < 0.015) supported weak、strong and strict invariance (see Table 6). Thus, the measurement invariance of the CES-D across gender among clinical sample was established.

### Difference test

In the clinical patients, females scored significantly higher than males on the score of AI (t = − 2.956, *p* < 0.01, Cohen’s d = 0.770) (Table 7). In the undergraduates, males scored significantly higher than males on the total score of the CES-D and scores of SC, IP (Total score: t = 2.033, *p* < 0.05, Cohen’s d = 0.074; SC score: t = 5.599, *p* < 0.01, Cohen’s d = 0.208; IP score: t = 4.092, *p* < 0.001, Cohen’s d = 0.150; PA score: t = 3.005, *p* < 0.05, Cohen’s d = 0.011;). Compared with the undergraduate group, the clinical group got significantly higher scores on total CES-D (t = − 32.274, *p* < 0.001, Cohen’s d = 2.127) and all five subscales (t: − 18.767 ~ − 31.676, all *p* < 0.001, Cohen’s d: 1.257 ~ 2.101) (Table 8).

**Table 7:** Comparison between Male and Female (Means±SD)

	Undergraduates					Clinical patients				
	male(*n* = 1764)	female (*n* = 1329)	t	*p*	Cohend’s d	male(*n* = 139)	female (*n* = 197)	t	*p*	Cohend’s d
DA	11.9 ± 3.2	11.8 ± 3.1	0.162	0.380	–	17.1 ± 4.0	17.9 ± 4.3	−1.706	0.093	–
SC	6.1 ± 1.8	5.7 ± 1.6	5.599	0.003	0.208	12.4 ± 3.2	12.7 ± 3.4	−0.889	0.375	–
AI	4.6 ± 1.5	4.8 ± 1.5	−3.757	0.870	–	7.4 ± 2.7	8.3 ± 2.2	−2.956	0.003	0.77
IP	2.9 ± 1.1	2.8 ± 1.0	4.092	0.000	0.150	4.7 ± 1.7	4.6 ± 1.7	0.303	0.762	–
PA	7.2 ± 2.4	7.0 ± 2.1	3.005	0.003	0.011	11.1 ± 2.6	11.1 ± 2.8	−0.121	0.904	–
CES-D	32.6 ± 7.9	32.1 ± 7.5	2.033	0.042	0.074	52.7 ± 11.4	54.6 ± 12.2	−1.446	0.149	–

**Table 8:** Comparison between Undergraduates and clinical patients (Means±SD)

	Undergraduates(*n* = 3093)	Clinical patients (*n* = 336)	t	*p*	Cohend’s d
DA	10.1 ± 2.8	17.6 ± 4.2	−31.676	0.000	2.101
SC	7.6 ± 2.2	12.6 ± 3.3	−26.845	0.000	1.782
AI	4.7 ± 1.5	7.9 ± 2.5	−25.805	0.000	1.552
IP	2.8 ± 1.1	4.6 ± 1.7	−23.636	0.000	1.257
PA	7.1 ± 2.3	11.1 ± 2.7	−18.767	0.000	1.594
CES-D	32.4 ± 7.8	53.8 ± 11.9	−32.274	0.000	2.127

## Discussion

The current study aimed to explore the best factor structure and measurement invariance of the Chinese version of the CESD among undergraduates and clinical patients. The CFA was conducted, suggesting that five-factor model was best suited in the two samples. Moreover, gender invariance was well established among undergraduates and clinical patients. To our knowledge, this was the first study to explore the measurement invariance of Chinese version of CESD across gender in clinical patients. Besides, The CES-D also showed acceptable internal consistency in the two samples.

The two-factor, three-factor, four-factor and five-factor models of the CES-D proposed in previous studies were all tested by CFA in the present study. In the original psychometric testing of the CES-D scale, Radloff proposed a four-factor structure comprising DA (depressed affect), PA (positive affect), SC (somatic/vegetative complaints), and IP (interpersonal problems) [8]. The current results found that the five-factor (Model E: DA, PA, IP, SC, and AI) showed the best fit. This five-factor model differs from the original four-factor model by changing the previous factor structure and proposing a new factor — alienation (items 10, 14, and 17). Alienation is a condition in social relationships reflected by a low degree of integration or common values and a high degree of distance or isolation between individuals, or between an individual and a group of people in a community or work environment. This particularly factors may impairments in interpersonal relationships. Previous study found that higher scores for thinking that others were out to harm or exploit them (alienation), the more likely participants were to experience a co-occurring mood disorder. School maladjustment in relations with teacher and peers and in learning activities had indirect effects through alienation and depression on students’ suicidal ideation [31].

Prior studies have shown that the original four-factor structure of the CES-D was not suitable for Asian population [15]. In addition, a recent study suggested that ethnic and cultural factors can lead to different CES-D factor structures [32]. The understanding of words or cultural differences may play an important role in different model structures. In addition, the population tested is may also play an important role. The current experiment included college student participants. The results may reflect the unique psychological characteristics, high level of education, and sensitivity of college students. This population is more likely to experience feelings of loneliness and alienation [32, 33].

The reliability and validity of the CES-D scale for was previously studied. It was proposed that the three-factor structure was the most suitable model. However, the five-factor model was not included in the study. In the current study, which included both the three- and five-factor model, the CFA on the five-factor structure showed the best fit. Accordingly, we concluded that the five-factor CES-D scale is an effective and reliable screening tool for depression.

Based on the five-factor model, we examined the gender invariance among among undergraduates and clinical patients. Our MGCFA confirmed good configural, weak, strong and strict invariance of the Chinese RRS-10 across gender in undergraduates sample. Configural equivalence is the precondition to test other equivalence. As the baseline model, the further equivalence test is the nested model produced by restricting the corresponding parameters on the basis of configural equivalence, only if the equivalence of the previous level is established, can the equivalence test of the next higher level be continued. In this study, the configural invariance of CESD was supported, so it can be used for the next step of equivalence test. Besides, the establishment of weak equivalence model shows that the CESD observation index and latent trait have the same meaning between men and women, that is to say, each item has the same unit between men and women. Moreover, the establishment of strong equivalence shows that the intercept of CESD is invariable between men and women, which means all CESD items have the same reference point in the two groups. Finally, strict equivalence is carried out on the basis of strong equivalence, and its establishment indicates that the measurement error variance is equivalent in different gender. Therefore, measurement invariance between males and females among undergraduates patients were achieved. In clinical samples, configural, weak, strong and strict invariance were also supported. Thus, the results of this study confirm that the Chinese CESD has strict equivalence, indicating that the scale is effective and interpretable between gender groups among undergraduates and clinical patients.

Since the CES-D has achieved measurement invariance across gender among undergraduates and clinical patients, this study further compared gender differences in CES-D and its subscale scores. The current study found that females scored significantly higher than males on the AI subscale in clinical patients. Besides, the current study also found that males scored significantly higher than females on the SC and IP subscale among college students. According to previous research, there are some sex differences in interpersonal problems [34]. For instance, boys are not good at talking, so girls are better than boys in speech skills [35]. About somatic complaints, Hyde found that the differences are demonstrated between boys and girls, boys are more physically and verbally aggressive than girls [36]. And they have higher domineering, controlling, independent behaviors and are more vindictive [35, 37]. In 2000, a research from American Psychiatric Association, illustrated boys exhibit higher rates of antisocial, narcissistic, obsessive compulsive, paranoid, and aggression-related disorders than girls. In sum, according to these reasons, boys will have higher scores on SC and IP.

College males experience more pressure than college females, in life pressure, personal ambition, studying, love, job hunting, earning money and interpersonal relationships [32, 35]. In addition, male college students consume more alcohol than female college students, leading to greater depression [38]. Moreover, studies have shown that in traditional Chinese culture, men are the economic backbone of the family and thus experience greater economic pressure [7].

While the current study provides valuable data on the factor structure and measurement invariance of the CESD, it is not without limitations. First, all participants were from the Changsha University and as such, the results may not fully reflect depression in college students in China. Second, a cross-sectional design was employed with no long-term follow-up. Third, Han students only were included in the sample, and therefore the results may not apply to minority students. Lastly, we only considered measurement invariance across genders, and thus, measurement invariance across other factors such as ages and religions, remains unknown.

## Conclusions

The CESD has good psychometric characteristics and measurement invariance across genders among clinical patients. The present study that the CESD may provide reliable and valid self-reported assessments of depression among Chinese undergraduates and clinical patients.

## Data Availability

The datasets generated and analyzed during the current study are not publicly available due to no permission from participants to share anonymized participant data publicly but are available from the corresponding author on reasonable request.
